# Retraction: Aurora kinase-C-T191D is constitutively active mutant

**DOI:** 10.1186/1471-2121-13-17

**Published:** 2012-06-26

**Authors:** Jabbar Khan, Sanaullah Khan, Sobia Attaullah, Ijaz Ali, Shahid N Khan

**Affiliations:** 1 Department of Biological Sciences, Gomal University Dera Ismail Khan, Dera Ismail Khan, Pakistan; 2 Institute of Genetics and Development, University of Rennes1, Rennes1, France; 3 Department of Zoology, Kohat University of Science and Technology, Kohat, Pakistan; 4 Department of Zoology, Islamia College Peshawar (A Public Sector University), University Campus, Jamrod Road, Peshawar, Khyber, Pakhtunkhwa, 25120, Pakistan; 5 Institute of Biotechnology and Genetic Engineering, Khyber Pakhtunkhwa University of agriculture Peshawar, Khyber, Pakhtunkhwa, Pakistan

## **Retraction**

The editors regretfully retract the article [1] by Jabbar Khan, Sanaullah Khan, Sobia Attaullah, Ijaz Ali, and Shahid Khan (BMC Cell Biology 2012, 13:8) due to significant overlap with previously published article “Overexpression of Active Aurora-C Kinase Results in Cell Transformation and Tumour Formation” by Jabbar Khan, Frédéric Ezan, Jean-Yves Crémet, Alain Fautre, David Gilot, Marine Lambert, Christelle Benaud, Marie-Bérengère Troadec, and Claude Prigent (PLoS ONE 6(10): e26512. doi:10.1371/journal.pone.0026512). We apologise to all affected parties for the inconvenience caused.
